# Emergency Hysterectomy in a Hemodynamically Unstable Patient: A Case of Uterine Leiomyosarcoma

**DOI:** 10.7759/cureus.11586

**Published:** 2020-11-20

**Authors:** Ermioni Tsarna, Lorena Kontou, Alexios Tsochrinis, Nestor Chavez, Nikolaos Georgopapadakos

**Affiliations:** 1 Obstetrics and Gynecology, General Hospital of Nikaia “Agios Panteleimon”, Nikaia, Piraeus, GRC

**Keywords:** hemodynamic instability, abnormal uterine bleeding, leiomyosarcoma, hysterectomy

## Abstract

Abnormal uterine bleeding (AUB) is a common gynecological complaint in reproductive aged women. In this case report, we present a case of emergency total hysterectomy performed in a hemodynamically unstable patient due to AUB. Based on pelvic ultrasound (US) and CT scan along with the prevalence of uterine smooth muscle tumors, leiomyomatous uterus was the most likely preoperative diagnosis. The histological examination of the surgical specimen revealed a leiomyosarcoma with coagulative necrosis, cellularity, mitotic index greater than 20 mitotic figures per 10 high-power-fields, and local invasion of the myometrium at the tumor’s stalk. A positron emission tomography (PET) scan was performed postoperatively. The results revealed multiple hypermetabolic secondary lesions at the lungs bilaterally, liver, vaginal cuff, peritoneal involvement, and a small lesion at the left rectus femoris muscle. Thus, tumor was classified as stage IVB uterine leiomyosarcoma according to the International Federation of Gynecology and Obstetrics (FIGO) staging. The patient was referred to an oncology center for chemotherapy and hormonal therapy. Uterine leiomyosarcomas are the most common uterine sarcomas, but remain a rare entity among uterine smooth muscle tumors. Notably, the US imaging of both leiomyosarcomas and other uterine smooth muscle tumors are practically indistinguishable. Thus, diagnosis is difficult to be established prior to surgical treatment. Overall, prognosis in case of leiomyosarcoma is poor, and tumor stage III/IV, tumor size greater than 10 cm, mitotic index greater than or equal to 20 mitotic figures per 10 high-power-fields, and reactive nuclei for Ki67 more than or equal to 10% are associated with shorter survival period. Reliable risk scores to stratify the risk of malignancy in case of leiomyomatous uterus and guide the timing of surgical treatment are totally lacking, and, thus, hindering earlier diagnosis of leiomyosarcoma and improved prognosis.

## Introduction

Abnormal uterine bleeding (AUB) is a common gynecological complaint, estimated to affect 3%-30% of reproductive aged women [1]. The large variation in prevalence estimates is attributed not only to true variation by age and country of origin, but also to regional patterns of accessibility to healthcare services [1]. The underlying causes of AUB are grouped into structural, which can be imaged, and nonstructural. Structural causes include endometrial polyps, adenomyosis, leiomyomas, malignancy, and endometrial hyperplasia, whereas nonstructural causes refer to coagulopathy disorders, ovulatory dysfunction, endometrial disorders, iatrogenic, and not otherwise specified [1]. Initial evaluation in case of AUB includes the following: full blood count, coagulation panel, speculum examination, bimanual pelvic examination, transvaginal, and transabdominal pelvic ultrasound (US).

## Case presentation

A 46-year-old Caucasian female premenopausal patient, gravity 2 parity 2 (G2P2), presented in the ED due to fever (up to 38.9°C), AUB since one month ago, fatigue, and dizziness. Apart from the fever, the patient’s vital signs were within normal range, although she looked pale.

Speculum examination revealed active uterine bleeding, characterized by foul smelling. Cervical inspection did not reveal any pathology, though it was technically difficult due to the menorrhagia. Nonetheless, there was no tenderness on cervical motion or fornix palpation bilaterally. In bimanual pelvic examination, a large uterus was palpated.

A transvaginal US scan was performed. A large anteverted leiomyomatous uterus was observed. The imaging of the ovaries was not possible due to the uterine size. There was no free liquid into the recto-uterine pouch.

To investigate the cause of the fever, a chest X-ray was performed, which did not reveal any pathology, along with a polymerase chain reaction (PCR) test for coronavirus disease 2019 (COVID-19), which was also negative. In addition, a urine sample was obtained for urine culture that showed urinary tract infection from *Escherichia coli* (>10^5 CFU/mL). A full blood count was also obtained, as well as basic biochemical tests were done. Hemoglobin was 4.4 g/dL and hematocrit was 16.5%. Platelets were elevated (522000/μL) in accordance with blood loss.

The patient was admitted to the hospital due to severe anemia in order to receive transfusions. Additionally, she was treated with micronized progesterone (200 mg x 3), ferric proteinsuccinylate (800 mg x 3), ampicillin/sulbactam (1 g/2 g x 3), and azithromycin (500 mg x1).

**Figure 1:**
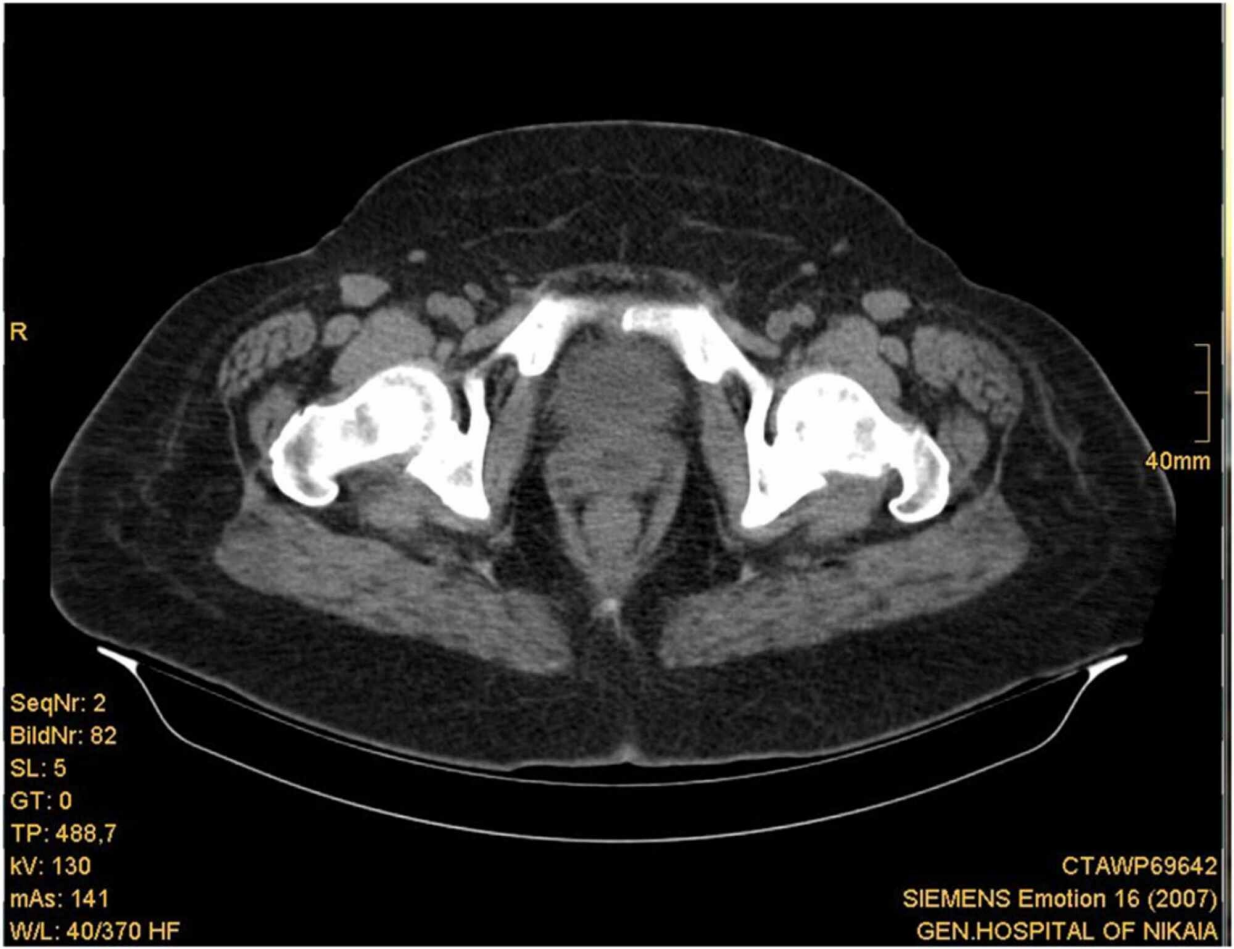
Abdominal CT scan showing the anatomic region of the uterine cervix.

**Figure 2:**
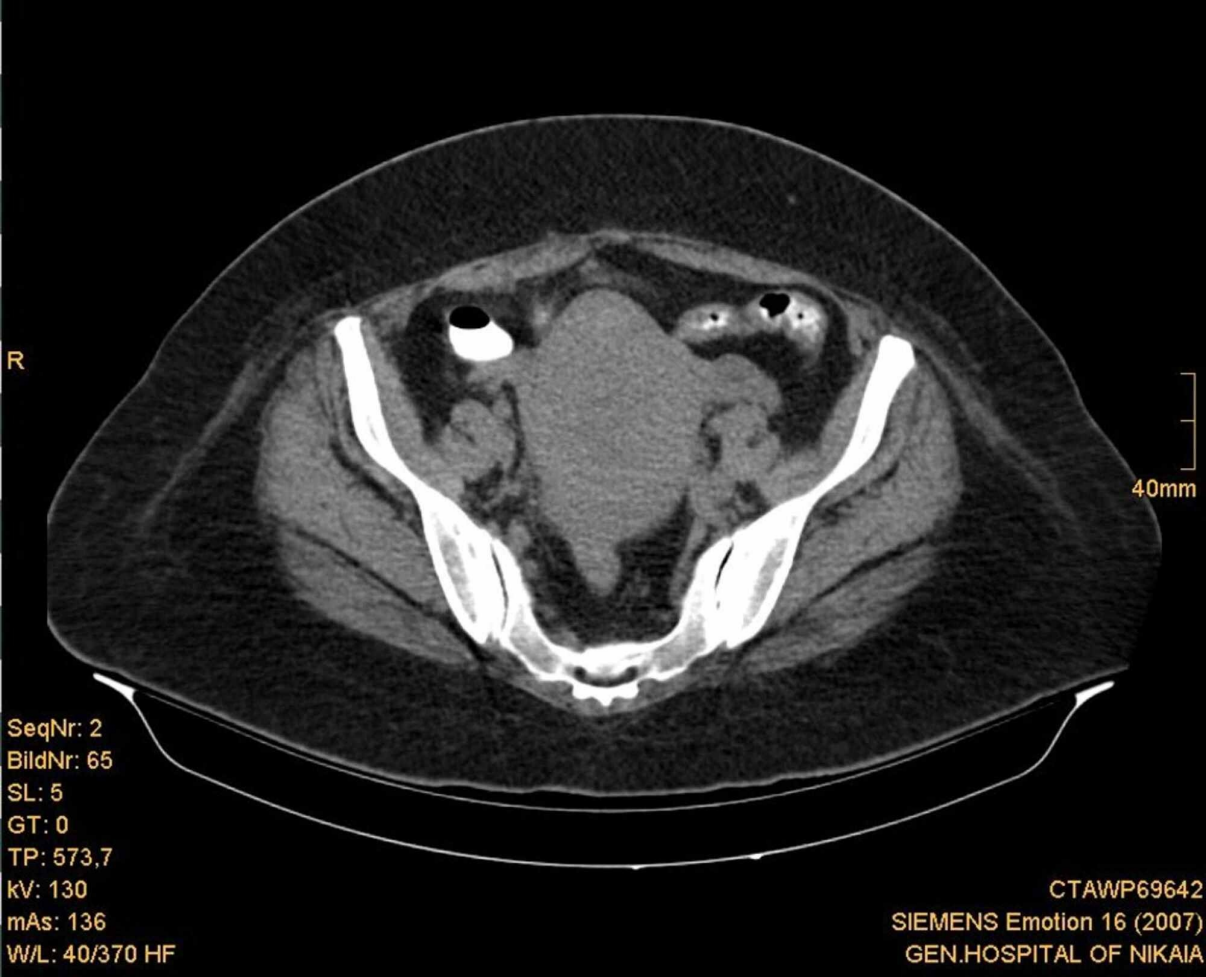
Abdominal CT scan showing the anatomic region of the uterine body.

**Figure 3:**
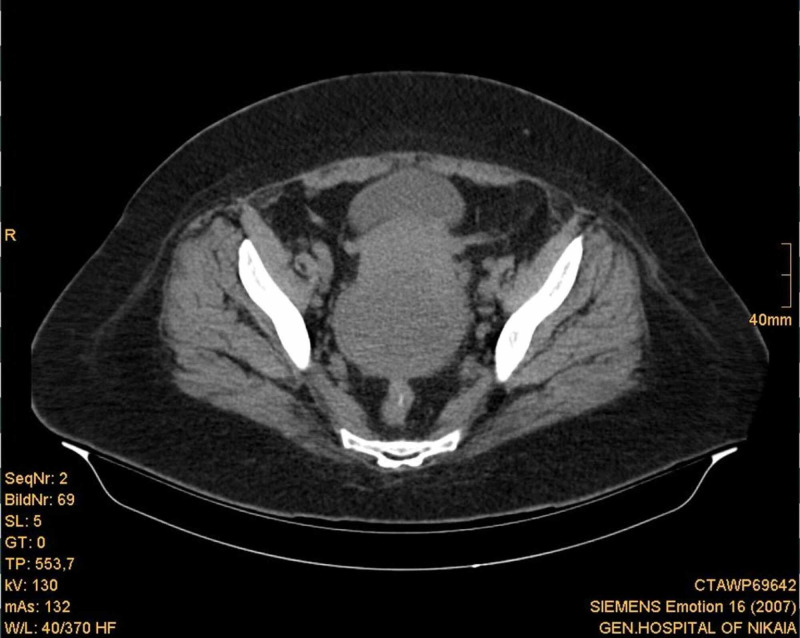
Abdominal CT scan showing the anatomic region of the uterine body.

An upper and lower abdominal CT scan with oral sodium amidotrizoate was performed, which revealed a large (approximately 7.5 cm diameter) hypodense lesion with clear boundaries in the anatomic area of the uterine cervix and body (Figures 1-3). There were no enlarged lymph nodes observed. Neoplasia was regarded as a probable diagnosis, although it was not possible to determine the origin of the lesion.

**Figure 4:**
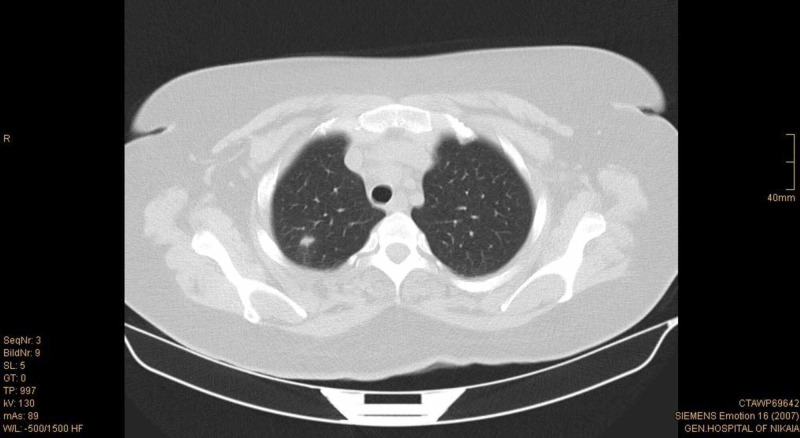
Chest CT scan that shows a nodule at the right upper pulmonary lobe.

**Figure 5:**
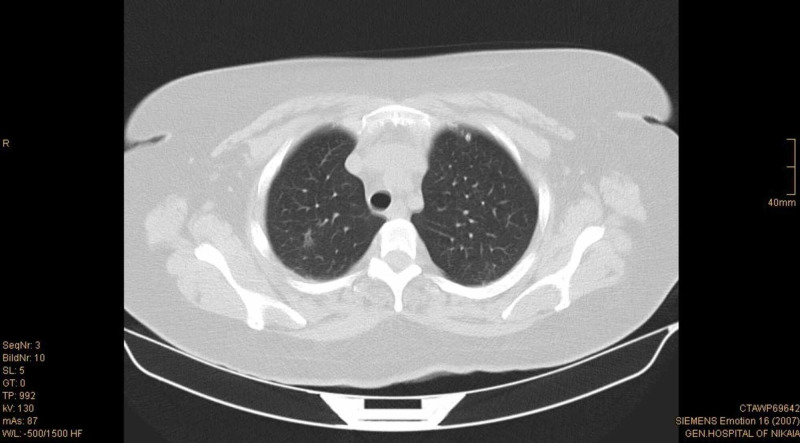
Chest CT scan that shows multiple small round nodules bilaterally.

A thoracic CT scan was also performed, which revealed multiple small round nodules bilaterally, without enlarged lymph nodes (Figures 4-5). The largest nodule was at the right upper lobe and had 1 cm diameter. Due to the chest CT findings, a pulmonologist’s expert opinion was sought. Based on patient’s past medical history of mycobacterium tuberculosis infection, the nodules were thought to have most likely infectious origin. However, neoplasia could not be ruled out from the differential diagnosis.

The patient was afebrile after 3 days of IV antibiotics. During her hospitalization, she received in total seven units of red blood cells (RBC) and three units of fresh frozen plasma (FFP). After one week she was discharged with hemoglobin 9.1 g/dL and hematocrit 30.3%. She was advised to undergo total hysterectomy in an oncology center rather than in a general hospital due to the suspicion of neoplastic disease.

At the sixth day from her discharge, the patient presented again in the ED due to fatigue, dizziness, tachycardia, and AUB, which was worse compared to any prior incidence. The full blood count revealed once again severe anemia. In particular, hemoglobin was 4.8 g/dL and hematocrit 16.3%. She was re-admitted for hemodynamic stabilization and transfusion.

After five days of hospitalization, and despite having received five units of RBC and two units FFP, the patient experienced an episode of loss of consciousness while she was trying to stand up from sitting position. Her hemoglobin was 6.3 g/dL and hematocrit 19.3%. After receiving three more units of RBC and one unit of FFP the patient was still hemodynamically unstable. She was unable to sit or stand up and she was tachycardic (120 beats per minute), despite all our efforts to support her with IV fluids and transfusions.

**Figure 6:**
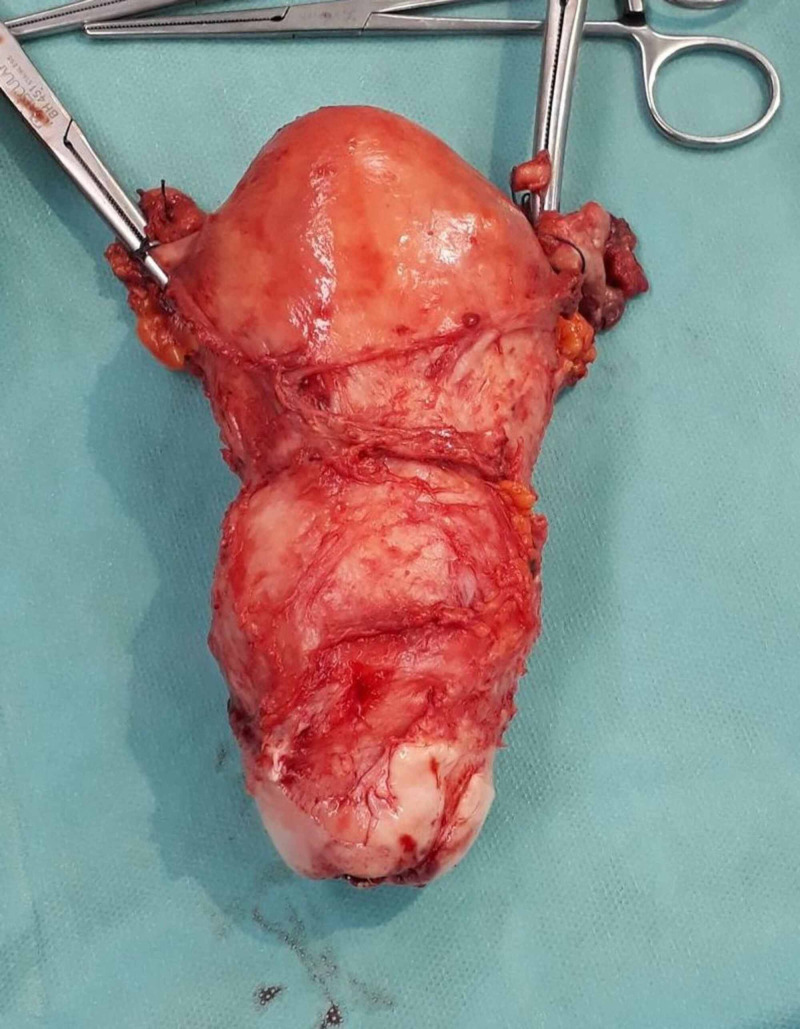
Surgical specimen includes the uterus en block with both fallopian tubes and ovaries.

**Figure 7:**
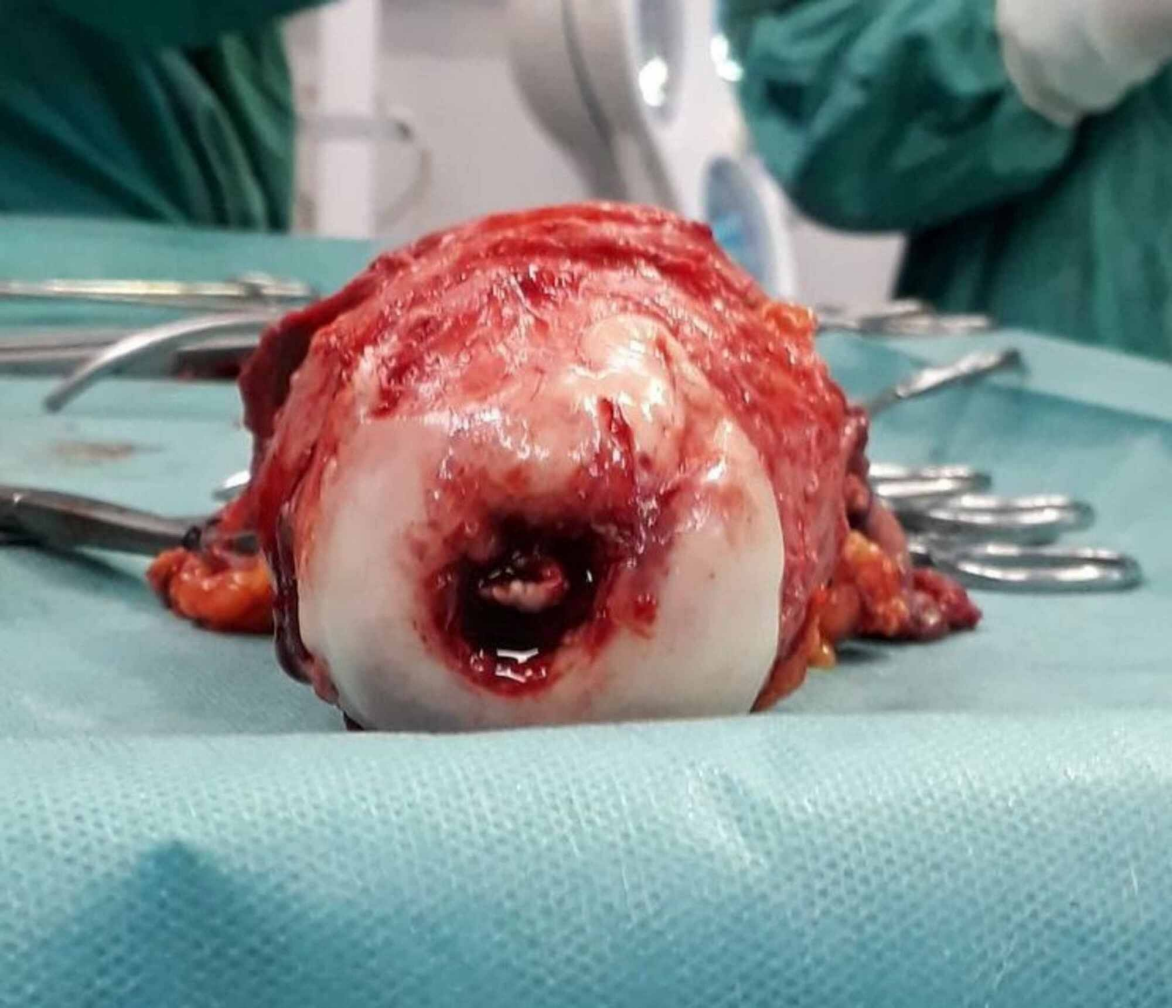
Surgical specimen showing the dilated uterine cervix.

**Figure 8:**
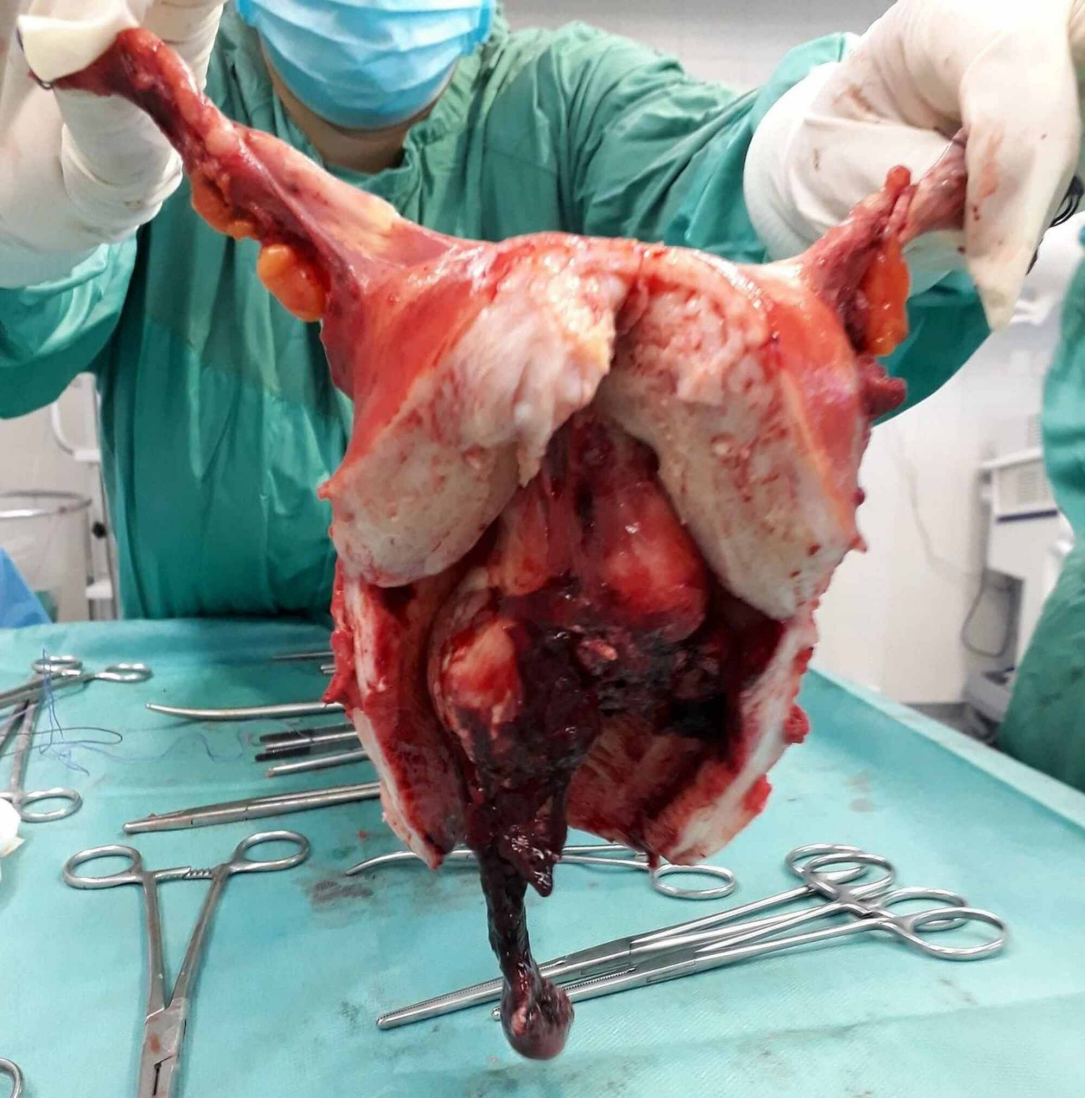
Surgical specimen showing the myometrial thickness and a polypoid intracavitary that is attached to the endometrium of the uterine fundus.

Due to the nonresponding hemodynamic instability, the patient was transferred to the operating theatre on an urgent basis with an aim to control menorrhagia. Initially, dilatation and curettage were performed. Interestingly, apart from endometrial tissues, solid material was also removed during curettage. Rapid histological assessment of the solid material was ordered and it did not reveal any findings compatible with neoplasia. Due to persistent uterine bleeding, we proceeded to emergency hysterectomy under general anesthesia. Via Pfannenstiel incision, uterus was removed en block with both fallopian tubes and ovaries (Figure 6). The uterine cervix was dilated (Figure 7), the myometrial thickness was 3.5 cm, the endometrial cavity was dilated, and a polypoid mass 8 cm x 4 cm x 6 cm was observed intracavitary that was attached to the endometrium of the uterine fundus (Figure 8). Extension of the Pfannenstiel incision to Maylard incision was not required intraoperatively. During the hysterectomy, the patient received three units RBC and two units FFP. The patient was discharged from the hospital on the third postoperation day. She was hemodynamically stable and in good general condition. Her hemoglobin was 7.7 g/dL and hematocrit 23.9%.

 

 

The histological examination of the surgical specimen revealed that the polypoid mass consisted of spindle cells with severe atypia and eosinophilic cytoplasm, which were arranged in bundles. Coagulative necrosis and cellularity were observed. Mitotic index was greater than 20 mitotic figures per 10 high-power-fields. The examination of the tumor’s stalk showed signs of local invasion of the myometrium. The results of the immunohistochemical panel are presented in Table 1 and were compatible with uterine leiomyosarcoma. In addition to the aforementioned, several small leiomyomas were also observed in the myometrium.

**Table 1:** Immunohistochemical panel.

Immunohistochemical marker	Result
Vimentin	Positive
Desmin	Positive
Estrogen receptors	Locally positive
CD 10	Locally positive
S100	Negative
C-kit	Negative
Actin	Negative
Caldesmon	Negative
Ki 67	Positive in >50% of neoplastic cells

**Figure 9:**
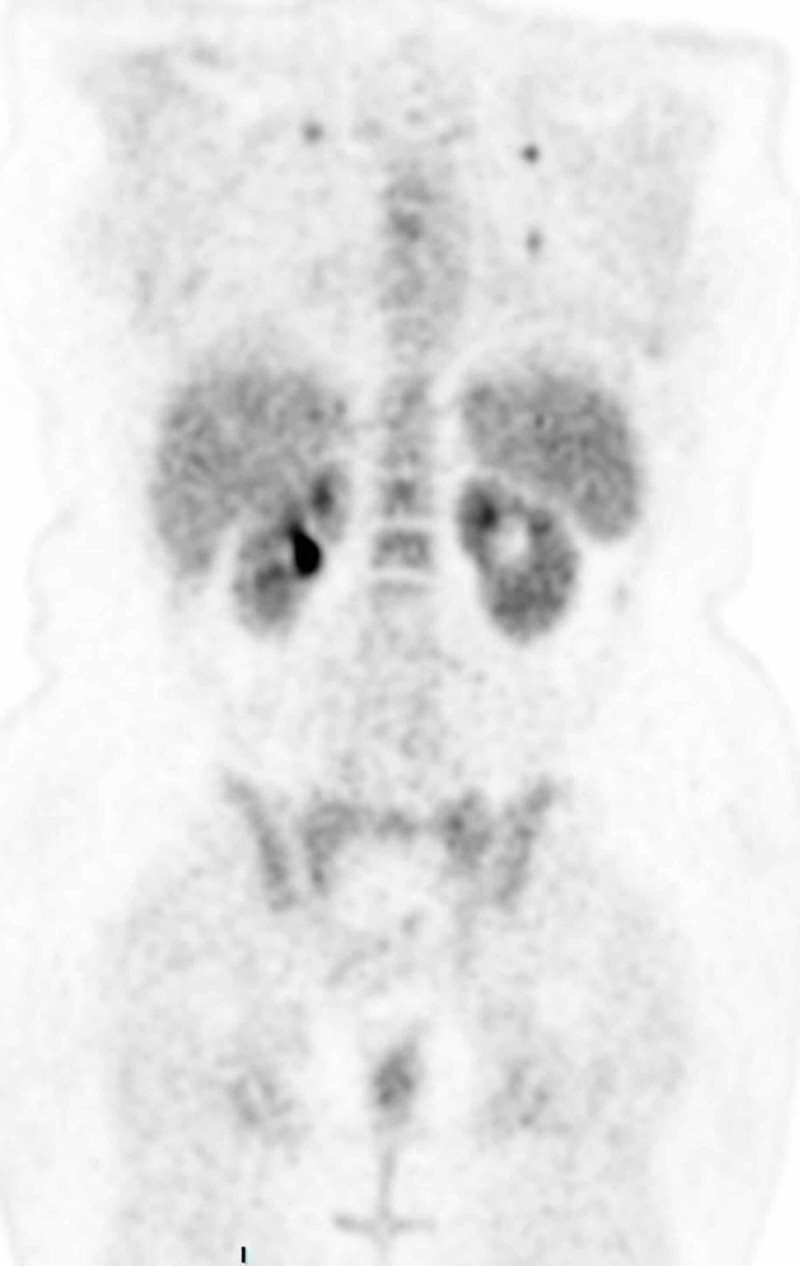
PET scan showing hypermetabolic secondary lesions at the lungs bilaterally. PET, positron emission tomography

**Figure 10:**
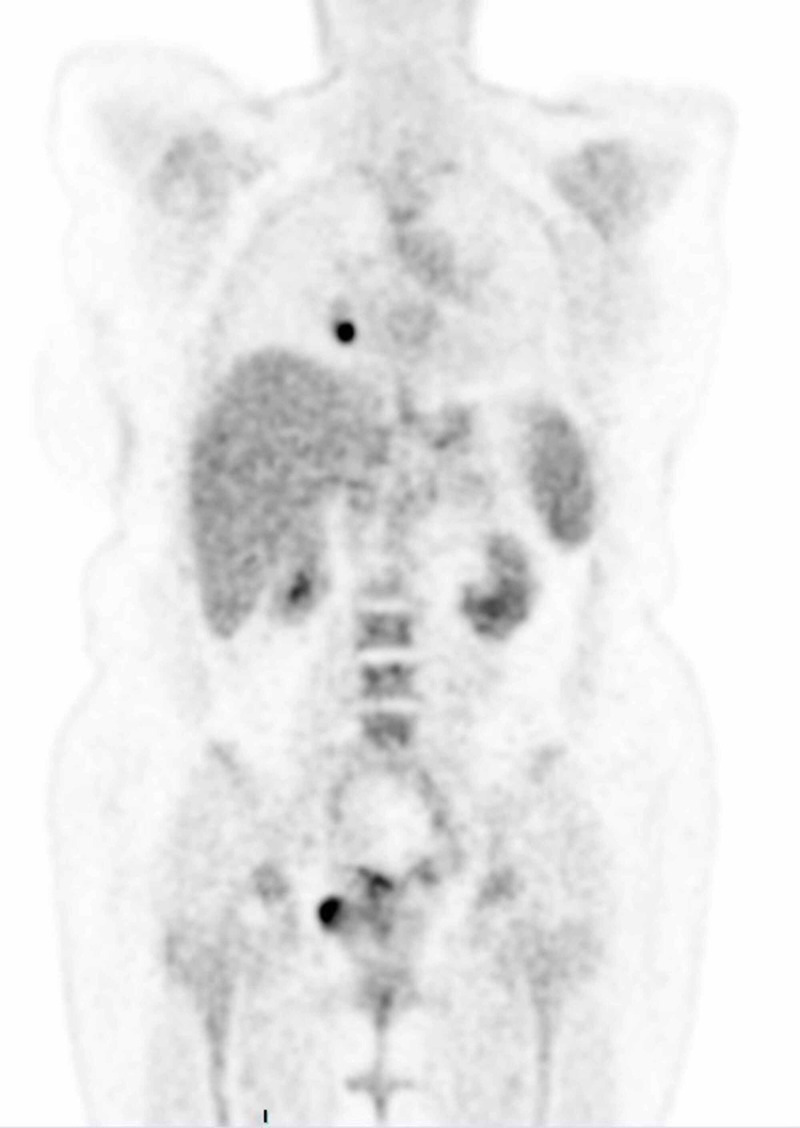
PET scan showing hypermetabolic pulmonary and liver secondary lesions. PET, positron emission tomography

**Figure 11:**
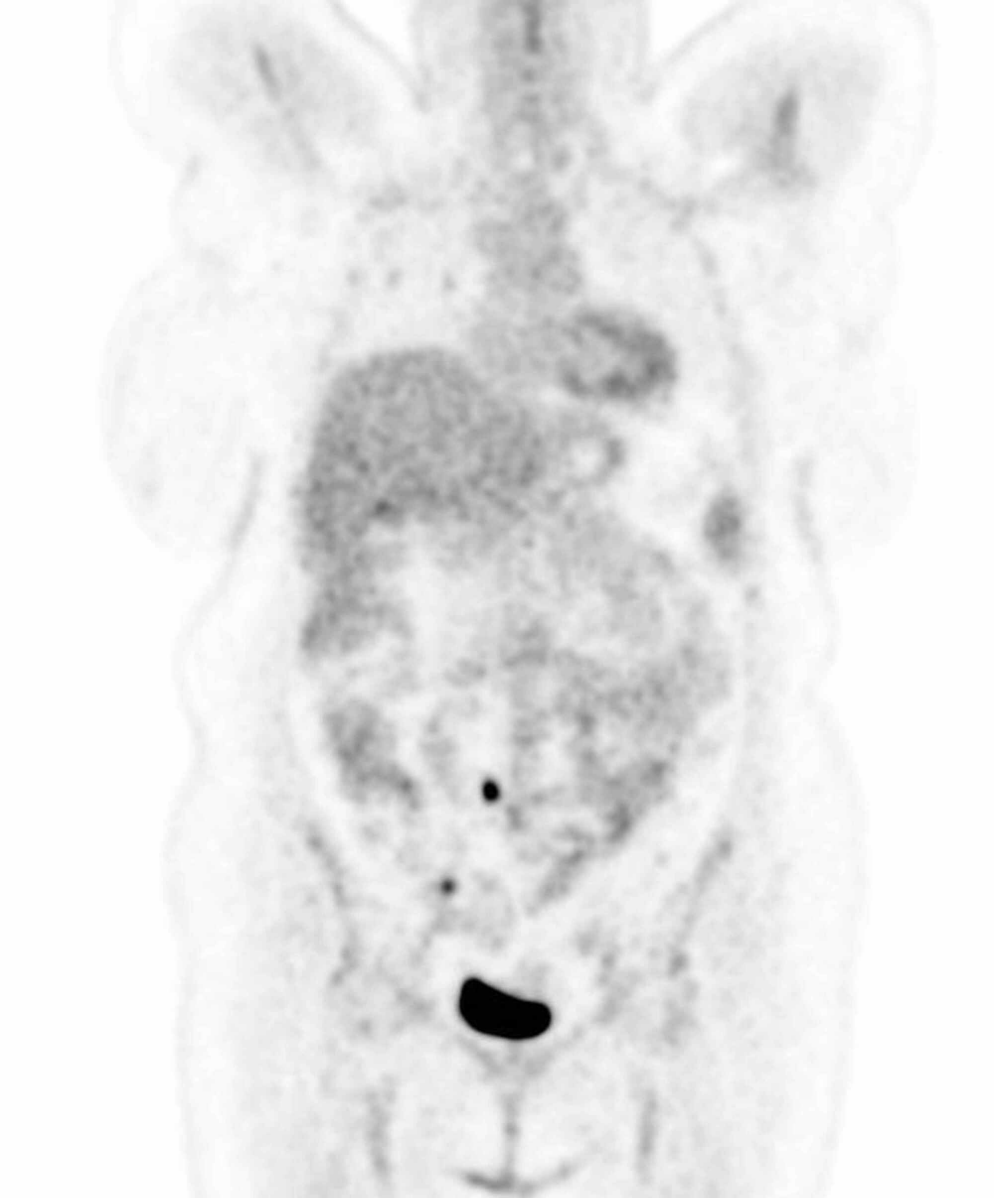
PET scan showing hypermetabolic secondary lesions at the peritoneum and the vaginal cuff. PET, positron emission tomography

**Figure 12:**
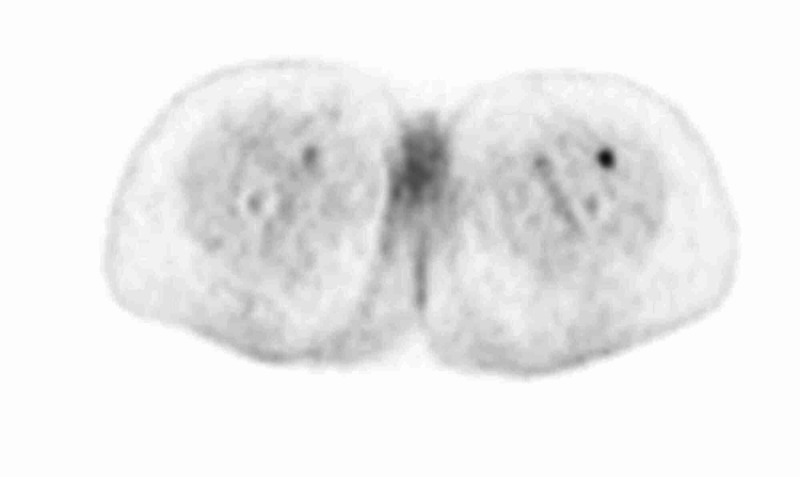
PET scan showing hypermetabolic secondary lesions at the left rectus femoris muscle. PET, positron emission tomography

After the establishment of uterine leiomyosarcoma diagnosis, a positron emission tomography (PET) scan was performed in order to determine the stage of the disease. The results revealed multiple hypermetabolic secondary lesions at the lungs bilaterally, liver, and vaginal cuff (Figures 9-11). Additionally, peritoneal involvement and a small lesion at the left rectus femoris muscle were reported (Figures 11-12). Thus, the definitive diagnosis was stage IVB uterine leiomyosarcoma, according to the International Federation of Gynecology and Obstetrics (FIGO) staging [2]. The patient was referred to an oncology center, and is currently treated with chemotherapy and hormonal therapy.

 

## Discussion

A case of uterine leiomyosarcoma in a hemodynamically unstable patient that required abdominal total hysterectomy on an urgent basis has been presented here. The diagnosis was established based on the histological assessment of the surgical specimen and further postoperative investigation revealed that it was stage IVB due to the presence of distant metastases.

Uterine sarcomas are rare gynecological malignancies. In particular, they represent only 1% of all malignancies of the female genital tract and 3%-7% of uterine malignancies [3]. They are classified into leiomyosarcomas, which are by far the most common among uterine sarcomas, endometrial stromal sarcomas, and undifferentiated sarcomas [2]. In the past, uterine carcinosarcomas were also classified under uterine sarcomas; however, based on their natural history and spreading pattern, they have been reclassified as endometrial carcinomas [2].

Uterine leiomyosarcomas account for 60% of all uterine sarcomas [2]. Nonetheless, only 1 in 800 uterine smooth muscle tumors prove to be leiomyosarcomas [4]. In pelvic US, leiomyosarcomas appear similar to the far more common leiomyomas, although size larger than 10 cm and progressively increasing size should raise the suspicion of malignancy [2]. The typical patient with uterine leiomyosarcoma is usually above 40 years old (55 years old on average at diagnosis), presents with AUB and occasionally with pelvic pain, and a pelvic mass is palpated in bimanual pelvic examination [3, 5]. In vast majority, leiomyosarcomas are sporadic; however, germline mutations in fumarate hydratase have been reported [2]. According to FIGO, uterine leiomyosarcomas are classified as stage I in case they are limited to the uterus, as stage II when the tumor extends beyond the uterus but is still in the pelvis, as stage III in case of abdominal invasion, and finally as stage IV when bladder or rectum is invaded or distant metastases are present [2]. Notably, metastases are mostly hematogenous rather than lymphatic.

Uterine leiomyosarcomas are associated with poor prognosis. Some 53%-71% of patients will experience a recurrence, most commonly in the lungs rather than the pelvis, five-year survival rate is reported between 15% and 25% across all stages, and the median survival is only 10 months [6-9]. Several studies have explored factors that associate with an unfavorable prognosis, but there has been poor consistency between them. Tumor stage III and IV, tumor size greater than 10 cm, mitotic index greater than or equal to 20 mitotic figures per 10 high-power-fields, and reactive nuclei for Ki67 more than or equal to 10% are thought to predict a poor prognosis [2].

## Conclusions

Uterine leiomyosarcomas are rare malignant tumors originating from the uterine smooth muscles that are associated with poor prognosis. Accurate diagnosis remains difficult to achieve prior to histological assessment of the surgical specimen from total hysterectomy, which is usually performed due to AUB on the grounds of leiomyomatous uterus. Notably, reliable risk scores to stratify the risk of malignancy in a woman with a leiomyomatous uterus and guide the timing of surgical treatment are totally lacking, posing an obstacle to earlier diagnosis of leiomyosarcoma and, thus, improved prognosis in these patients.
